# Algorithm for pre-emptive glycopeptide treatment in patients with haematologic malignancies and an *Enterococcus faecium* bloodstream infection

**DOI:** 10.1186/2047-2994-2-24

**Published:** 2013-09-11

**Authors:** Xuewei Zhou, Jan P Arends, Lambert FR Span, Alexander W Friedrich

**Affiliations:** 1 Department of Medical Microbiology, University of Groningen, University Medical Center Groningen, Groningen, The Netherlands; 2 Department of Haematology, University of Groningen, University Medical Center Groningen, Groningen, The Netherlands

**Keywords:** *Enterococcus faecium*, Haematologic patients, Risk factors, Glycopeptides, Antibiotic stewardship

## Abstract

**Introduction:**

Nowadays *Enterococcus faecium* has become one of the most emerging and challenging nosocomial pathogens. The aim of this study was to determine risk factors in haematology patients who are at risk of an *Enterococcus faecium* bloodstream infection (BSI) and should be considered for pre-emptive glycopeptide treatment. With these identified risk factors a prediction model can be developed for clinical use.

**Methods:**

Retrospectively clinical and microbiological data in 33 patients with an *E. faecium* BSI were compared to 66 control patients during a 5-year period at the haematology ward. Multivariate logistic regression was used to explore the independent risk factors and a prediction model was developed to determine the risk of an *E. faecium* BSI.

**Results:**

*E. faecium* BSIs were found to be associated with high mortality rates. Independent risk factors for *E. faecium* BSI were colonization with *E. faecium* 30 days prior to blood culture (OR 5.71; CI 1.7-18.7), combination of neutropenia and abdominal focus (4.37; 1.4-13.4), age > 58 years (4.01; 1.3-12.5), hospital stay prior to blood culture > 14 days (3.55; 0.98-12.9) and CRP (C-reactive protein) level >125 mg/L (4.37; 1.1-10.2).

**Conclusion:**

Using data from this study, risk stratification for the development of an *E. faecium* BSI in patients with haematological malignancies is possible. Pre-emptive treatment should be considered in those patients who are at high risk. Using a prediction model as designed in this study, antibiotic stewardship in terms of prudent use of glycopeptides can be improved and might be helpful in controlling further spread of VRE (vancomycin resistant enterococci).

## Introduction

*Enterococcus faecium* has become one of the most important, emerging and challenging nosocomial pathogens [1]. It is a difficult to treat pathogen due to intrinsic resistances to cephalosporins, aminoglycosides (low-level resistance), clindamycin and trimethoprim-sulfamethoxazole [2]. Moreover, it has the ability to easily acquire virulence or antibiotic resistance genes trough transfer of plasmids, chromosomal exchange or mutation [3].

Due to the resistance of multiple antibiotics, the treatment of choice in serious *E. faecium* infections is glycopeptides. However, prudent use of vancomycin is needed as it is associated with an increased risk for VRE infection and colonization [4]. The emergence of VRE has been reported one to two decades ago in the United States [5]; more recently alarming reports are now coming from many countries in Europe [6].

Several studies have pointed out the existence of two subpopulations of *E. faecium*: commensal/community-associated (CA) strains and clinical or hospital associated (HA) strains, whereas the latter is also referred as the clonal complex 17 (CC-17) group [7]. These HA/CC-17 strains are associated with ampicillin resistance; the rise and replacement of *E. faecium* as the predominant enterococcus species are especially due to these strains [8].

A predominant part of the nosocomial *E. faecium* bloodstream infections concerns patients with haematologic malignances who are immunocompromised by their severe disease and intensive treatment. Whereas it often is debated whether to treat *E. faecium* as a real pathogen, several studies have shown high morbidity and mortality rates for enterococcal bacteremia (mortality rates ranging from 25% to 51%), especially in immunocompromised patients [9-11]. Moreover, the mortality rates increases with inappropriate antimicrobial therapy [12].

After coagulase negative staphylococci (CoNS), streptococci and *Escherichia coli* (*E. coli*), *E. faecium* is the most predominant species isolated among blood cultures at the haematology unit of our hospital. Compared to other pathogens such as CoNS, *E. coli, Pseudomonas aeruginosa (P. aeruginosa)* and streptococci which remained stable or decreased, *E. faecium* increased for the periods 1998–2006 (3.1%) and 2007–2010 (12.8%) which is 4.1 times more.

Since patients with haematologic malignancies are highly prone to infection, prophylactic antibiotics are used to prevent and reduce any risk of infection. In our haematology ward penicillin and ciprofloxacin or co-trimoxazol or colistine or tobramycin (orally) are used depending on the resistance pattern of bacteria found in surveillance cultures. In case a haematology patient presents with neutropenic fever or other clinical signs of infection, blood cultures are taken and empirical broad-spectrum antibiotic treatment is started, which is piperacillin-tazobactam.

Glycopeptides are not recommended as a standard part of the initial antibiotic regimen for fever and neutropenia. Moreover, as noted earlier, for the further prevention and control of VRE it is necessary to control the use of glycopeptide antibiotics. At this moment, glycopeptides are only added in case of a positive blood culture with *E. faecium* or oxacillin resistant CoNS*.* However blood culture results and their susceptibilities are only available after one or more days after blood samples are drawn.

Therefore the aim of this study is to identify possible risk factors in those haematology patients who are at high risk of *E. faecium* bloodstream infection in order to develop a prediction model for clinical stringent use. This can be useful in the decision of pre-emptive therapy with glycopeptides together with the initial empirical antibiotic treatment at the moment a blood culture is taken.

## Methods

### Study design and population

**Figure 1:**
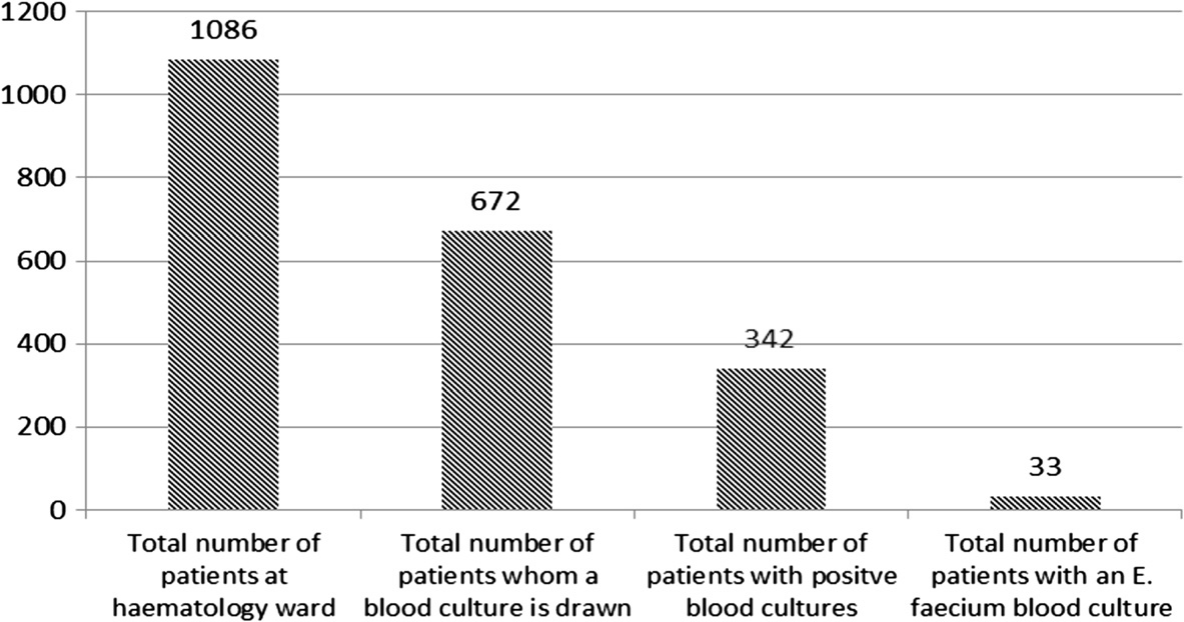
**Patients at the haematology ward of the UMCG during the period September 2005-September 2010: Thirty-three of the patients with positive blood cultures (672) had an *E. faecium *blood culture (~5%).**

The University Medical Center Groningen (UMCG) is a 1300-bed tertiary center and has a 27-bed haematology ward. This ward has four 4 patient rooms, one double room and nine private rooms. Patients were identified by a search of the laboratory electronic database for all blood cultures between September 2005 and September 2010 from the haematology ward. In this period a total of 1086 patients were admitted to the haematology ward of whom 672 blood cultures were taken. (Figure 1) Case patients were identified by a search for all blood cultures positive for *E. faecium*. Of each patient with an *E. faecium* blood culture, the first positive blood culture was selected: a total of 33 patients with *E. faecium* blood cultures were identified. For the main purpose of our study, (an algorithm to decide whether or not to add glycopeptides to the initial empirical antibiotic therapy at the moment a blood culture is taken) we choose to use a selection of all the patients of which a blood culture was taken (positive as well as negative), except those with *E. faecium* blood culture (*n =* 672-33 = 639). After all, this whole group had the same grounds to obtain a blood culture at the (retrospective) moment the blood culture was drawn. This would also be the case in prospective situations where this algorithm could be applied on. A total of 66 control patients were randomly selected: first a patient was randomly selected; subsequently a blood culture was randomly selected. Patients were not matched for age or sex.

### Data collection

Patient data were gathered by reviewing hospital electronic records and stored hard-copy records. The date the blood culture was taken was chosen as day 0 and from that point all data were reviewed all data retrospectively. Clinical data collected included information of underlying disease, admission status, co-morbidities, neutropenia, C-reactive protein (CRP) levels, fever and signs of organ failure prior to blood culture. Microbiological data collected included clinical source of infection, information about *E. faecium* colonization and antibiotic use 30 days prior to positive blood culture. If a patient had diarrhea, records were also reviewed for *Clostridium difficile*. Antibiotic susceptibility patterns, presence of polymicrobial bacteremia and positive galactomannan tests were gathered. Antibiotic treatment with vancomycin or teicoplanin for *E. faecium* bacteremia was evaluated. Outcomes were measured by need of ICU admission and mortality at 7 and 30 days after blood culture.

### Clinical notifications and definitions

During the retrospective study period, blood cultures were drawn for neutropenic fever or other clinical signs for infection. Fever was defined as temperature >38.5°C or > 38°C for 24 hours was a reason for further examination. An absolute neutrophil count below 0.5 × 10^9^/L was defined as neutropenia. For organ failure the following definitions were used: renal failure was defined as creatinin >176 μmol/L, hepatic failure as bilirubin >43 mmol/L and pulmonary failure as bilateral lung infiltrates or signs of acute respiratory distress syndrome (ARDS). These definitions were according to guidelines used for defining organ failure in severe sepsis [13]. Polymicrobial infection was defined as a micro-organism other than *E. faecium* within ± 7 days of the blood culture. For the controls it was defined as an additional micro-organism within ± 7 days of a positive blood culture. In this definition less pathogenic micro-organisms such as CoNS, *Corynebacteriae*, *Micrococcus* spp. and *Bacillus* spp. as an additional micro-organism were excluded.

### Infection prevention regimen haematology ward

At the haematology ward of our hospital, selective decontamination of the digestive tract (SDD) is performed in patients with an (expected) reversible neutropenia or increased risk of infection. The implementation is as follows: surveillance cultures from faeces, throat and urine at admission day, then once a week only faeces and throat cultures during the duration of neutropenia. Penicillin (to prevent streptococcal sepsis) and ciprofloxacin or cotrimoxazol or colistine or tobramycin (orally) are used as prophylactic antibiotics depending on the resistance pattern of surveillance cultures. Amphoterin B, nystatin or fluconazole are given orally as antifungal therapy. The choice of empirical antibiotic therapy is piperacillin-tazobactam.

Screening for *E. faecium* in this period was done on BME(G) agar plates. This contained Meropenem 64 mg/L, Oxacillin 10 mg/L, Amphotericin-B 20 mg/L and esculin. Hereby we screened for ampicillin resistant *E. faecium* (HA *E. faecium*). From January 2007 these agar plates also contained gentamicin 128 mg/L since there was an increase of high level gentamicin resistant *E. faecium* in our hospital from that time period.

### Identification and susceptibility testing

Blood cultures were performed using the BACTEC system (Becton Dickinson™). Further determination and susceptibility testing were performed for gram positive streptococci that were catalase negative and PYR positive. As for *E. faecium* surveillance cultures, only colonies that grew on the BMEG plates with black borders were further determined. Species were identified using the VITEK®2 System (BioMérieux™) or API20 Strep System (BioMérieux™). Subsequently antimicrobial susceptibility testing was performed using the VITEK®2 System or disk diffusion tests respectively.

### Statistical analyses

Statistical analyses were performed using SPSS for Windows, rel 18.0. Univariate analyses were performed using the Fisher’s exact or Chi-square methods for categorical variables. The Student’s t-test or Mann–Whitney *U*-test was used for the continuous variables. Results with a *p*-value of ≤0.05 were considered to be statistically significant. All *p*-values are two-tailed. Significant variables were used in the multivariate logistic regression.

### Deriving prediction model from a nested case–control design

To overcome the overestimation of risks because of overrepresentation of cases, we choose to perform a nested case–control design where the cases represent 5% and controls 95% of the whole population (Figure 1). Therefore the following factor to the intercept of the logistic regression model is added: c = ln (q_0_/ (1-q_0_)), whereas q_0_ is the true prevalence of the diseases in the population. With this correction the risk of an individual to get the disease can be estimated by the formula e^β0+c+ β1X1+…βkXk^ / 1 + e ^β0+c+ β1X1+…βkXk^. In this formula, β_0_ is the intercept from the linear regression equation, β_1_/β_k_ is the regression coefficient derived from the multivariate logistic regression and X_1_/X_k_ is the value of the predictor. In this study, q0 is the prevalence of patients with an *E. faecium* blood culture. Since we were only interested in those patients of whom a blood culture is drawn, c = ln (0.05/ (1–0.05)) = −2.94. Controls should be a random selection representative of the population [14] which is the case since we randomly selected the 66 control patients.

## Results

### Patients

A total of 99 patients were evaluated: 33 cases (*E. faecium*) and 66 controls. Characteristics of the 66 controls showed the following blood culture results: *E. coli* (n = 4), *Streptococcus viridans* (n = 2), CoNS (n = 4), *Corynebacterium* spp. (n = 1) and no growths (n = 55). Comparisons of the demographic and clinical data are presented in Table 1. There were no significant differences between type or status of disease. Patients with *E. faecium* bacteremia were associated with higher age and longer hospitalization days prior to blood culture as well as one year before admission. They were also associated with severe and longer duration of neutropenia, longer duration of fever and higher CRP levels at time of blood culture withdrawal. Penicillin and quinolones as a part of the SDD regimen and piperacillin-tazobactam as empirical broad-spectrum antibiotics were the most frequently used antibiotics; however this did not differ between the two groups. Only “other” antibiotics were more frequently given in the *E. faecium* group. This was mainly colistine, a polymixin antibiotic, though colistine use alone was not significant.

**Table 1:** **Comparison of demographic and clinical characteristics of cases (*E. faecium *) and controls**

**Demographics**	**Cases (*n*** **= 33)**	**Controls (*n*** **= 66)**	***p*-value**
**Male gender**	18 (68.2%)	45 (54.5%)	0.184
**Age, mean ± SD, years**	58.0 ± 11.3	52.2 ± 9.1	0.008
**Type of malignancy: **^**a**^			0.378
Leukaemia (AML, MDS, ALL) for chemotherapy	19 (57.6%)	28 (42.6%)	
Leukemia for allogeneic stem cell transplantation	2 (6.1%)	2 (3.0%)	
Lymphoma’s, Kahler, CLL and others undergoing autologous stem cell transplantation	6 (18.2%)	17 (25.8%)	
Lympfhoma’s, Kahler, CLL not undergoing autologous stem cell transplantation	6 (18.2%)	19 (28.8%)	
**Status of disease:**			
Remission	9 (27.3%)	11 (16.7%)	0.215
Not in remission ^b^	24 (72.7%)	55 (83.3%)	0.215
Relapse	7 (21.2%)	14 (21.2%)	1.000
**Reason for admission:**			0.476
Infection	4 (12.1%)	13 (19.7%)	
Chemotherapy	21(63.6%)	34 (51.5%)	
Stem cell transplantation ^c^	8 (24.2%)	19 (28.8%)	
**Length of hospital stay:**			
Length in days prior to positive blood culture, median (range)	21 (2–52)	13.5 (1–84)	0.007
Length in days 1 year before admission, median (range)	43 (6–131)	24 (1–133)	0.018
**Signs of organ failure: **^**d**^			
Renal (creatinine > 176 μmol/L)	2 (6.1%)	3 (4.5%)	0.746
Hepatic (bilirubin >34 mmol/L)	2 (6.1%)	0 (0.0%)	0.109
Lung (bilateral lung infiltrates)	4 (12.1%)	10 (15.2%)	0.769
**Days of fever, median (range) **^**d**^	2 (0–7)	0 (0–6)	0.001
**Neutropenia:**			
Neutropenia <0.1 × 10^9^/L ^e^	20 (60.6%)	19 (28.8%)	0.002
Neutropenia <0.5 × 10^9^/L ^e^	28 (84.8%)	28 (42.4%)	<0.001
Neutropenia <2.0 × 10^9^/L ^e^	29 (87.9%)	39 (59.1%)	0.004
Duration of neutropenia <0.5 × 10^9^/L prior to blood culture, median (range)	8.0 (0–27)	0.0 (0–26)	<0.001
**CRP (C-reactive protein in mg/L):**			
Levels 7 days prior to blood culture, median (range)	26 (3–263)	47 (5–347)	0.07
Levels at time of blood culture, median (range)	188 (7–288)	108 (3–426)	0.006
At time of blood culture CRP >125 mg/L	23 (69.7%)	24 (36.4%)	0.002
**Antibiotic therapy at time of blood culture and/or 30 days before:**			
Penicillins	24 (72.7%)	40 (60.6%)	0.234
Cotrimoxazole	12 (36.4%)	18 (27.3%)	0.353
Quinolones	25 (75.8%)	51 (77.3%)	0.866
Cephalosporins	6 (18.2%)	4 (6.1%)	0.079
Carbapenems	6 (18.2%)	5 (7.6%)	0.113
Others ^f^	19 (57.6%)	16 (24.2%)	0.001

^a^*AML*  acute myeloid leukaemia, *MDS*  myelodysplastic syndrome, *ALL*  acute lymphoblastic leukaemia, *CLL*  chronic lymphoblastic leukaemia. ^b^Including patients partially in remission. ^c^Allogeneic as well as autologous stem cell transplantation. ^d^At the day of blood culture till 7 days prior to blood culture. ^e^At the day of blood culture withdrawal ^f^colistin, tetracyclin, macrolides, aminoglycosides, metronidazole.

### Microbes

From the 33 cases, fourteen patients (42.4%) had a single blood culture, nineteen (57.6%) had more than one blood culture and 11 (33.3%) had more than two blood cultures. All *E. faecium* blood isolates were resistant to amoxicillin. No VRE strains were identified in this study. High-level gentamicin resistance (HLGR) was found in 19 (57.6%) of the 33 *E. faecium* blood isolates. Three of the 19 patients with HLGR *E. faecium* also had low level gentamicin resistant *E. faecium* in their blood cultures (multiple blood cultures).

Comparisons of the microbial data are presented in Table 2. Polymicrobial infections were found in 9.1% of the cases compared to 1 (1.5%) in the control group (*p* = 0.107). Pathogens isolated were *Clostridium perfringens*, *Pseudomonas aeruginosa*, and Streptococcus species. Three case patients (9.1%) had a positive Galactomannan compared to 2 (3.0%) in the control group (*p* = 0.330).

**Table 2:** **Comparison of the microbiological characteristics of cases (*E. faecium *) and controls**

	**Cases (*n*** **= 33)**	**Controls (*n*** **= 66)**	***p*-value**
Colonization with *E. faecium*^a^			
7 days prior to blood culture	13 (39.4%)	10 (15.2%)	0.007
30 days prior to blood culture	19 (57.6%)	14 (21.2%)	<0.001
90 days prior to blood culture	21 (63.6%)	16 (24.2%)	<0.001
Number of faeces cultures with *E. faecium* 30 days prior to blood culture, median (range)	1 (0–8)	0 (0–6)	<0.001
Type of blood culture			
Polymicrobial ^b^	3 (9.1%)	1 (1.5%)	0.107
Galactomannan	3 (9.1%)	2 (3.0%)	0.330
Clinical source of infection			
Abdominal focus: abdominal pain and/or diarrhea	25 (75.8%)	29 (43.9%)	0.003
Abdominal pain	9 (27.3%)	11 (16.7%)	0.215
Diarrhea	23 (69.7%)	26 (39.4%)	0.004
Mucositis	13 (39.4%)	18 (27.3%)	0.220
Lungs			
Coughing and/or sputum	8 (24.2%)	15 (22.7%)	0.866
Radiological proof of pneumonia or lung infiltrates	4 (12.1%)	14 (21.2%)	0.269
Ear Nose Throat	1 (3.0%)	2 (3.0%)	1.000
Skin	7 (21.2%)	19 (28.8%)	0.419
Urinary infection	1 (3.0%)	9 (13.6%)	0.158

^a^In faeces culture, part of the SDD regimen. ^b^Within ± 7 days, less pathogenic micro-organisms (coagulase-negative staphylococci, corynebacteria, micrococcus spp. and bacillus spp.) are excluded.

An abdominal focus was found to be associated with *E. faecium* bacteremia (*p* = 0.003) of which diarrhea appeared to be most distinct variable. Only one patient with an *E. faecium* BSI had a positive *Clostridium difficile* toxin test (no *C. difficile* in stool culture) at time of diarrhea. This was two days prior to the positive blood culture, together with a positive *E. faecium* faeces culture, though this patient was already colonized with *E. faecium* for several weeks.

Patients with *E. faecium* BSI were more often detected to be colonized with *E. faecium* prior to blood culture (*p* = <0.001). A total of twenty-one patients (63.6%) were colonized with *E. faecium* prior to the positive blood culture with a median of 1 (range 0–8), compared to 24.2% in the control group with a median of 0 (range 0–6). Twelve patients (36.4%) were not found to be colonized with the surveillance cultures. However, nine of these twelve patients had a blood culture with low level gentamicin resistant *E. faecium*. Seven of these twelve patients (58.3%), had a positive faeces culture with *E. faecium* after all within 30 days after positive blood culture; five with high level gentamicin resistant *E. faecium*, two with low level gentamicin resistant *E. faecium*. The majority of the patients (69.7%) were still colonized up to 30 days after the first positive blood culture. This includes both patients that were already colonized and patients who had a positive culture with *E. faecium* within 30 days after their positive blood culture.

### Outcomes and treatment

Both groups had an equal antibiotic treatment for piperacillin-tazobactam as well as for glycopeptide treatment at time of blood culture withdrawal. (Table 3) Patients with an *E. faecium* BSI were more often admitted to the ICU after the positive blood culture. Reasons for ICU admissions were predominantly sepsis, mostly with an abdominal focus (abdominal sepsis). The 7-day mortality as well as the 30-day mortality were significantly higher in patients with *E. faecium* BSI compared to the control group (30.3% vs 4.5%; *p* = 0.001 and 39.4% vs 10.6%; *p* = 0.001 respectively). All 10 patients with *E. faecium* BSI that died within 7 days after their last positive culture were diagnosed with sepsis or severe infection, six of them (60%) had an clear abdominal focus (abdominal sepsis). Another three patients died after 30 days, one diagnosed with a septic shock, the other two patients had multiple diagnoses.

**Table 3:** **Comparison of outcome and antibiotic treatment of cases ( *E. faecium *) and controls**

	**Cases (*n*** **= 33)**	**Controls (*n*** **= 66)**	***p*-value**
Piperacillin-tazobactam treatment at time blood culture is drawn and/or 30 days before	22 (66.7%)	42 (63.6%)	0.766
Vancomycin/teicoplanin treatment at time of blood culture withdrawal	4 (12.1%)	8 (12.1%)	1.000
ICU admission till 7 days after positive bloodculture	5 (15.2%)	1 (1.5%)	0.015
Mortality^*^			
At 7 days	10 (30.3%)	3 (4.5%)	0.001
At 30 days	13 (39.4%)	7 (10.6%)	0.001

^*^After last positive blood culture with *E. faecium.*

More detailed data considering antibiotic treatment in patients with an *E. faecium* BSI including mortality rates are presented in Table 4. Only 4 patients (12.1%) received glycopeptide treatment at time of blood culture withdrawal. Three of them had an empirical treatment and one received treatment because of an earlier proven CoNS infection. After 24 hours a total of 19 patients (57.6%) received glycopeptide treatment. Of these 19 cases, four were empirically treated upfront because of septic profile, two cases because of a CoNS infection and 13 cases recommended by the medical microbiologist because of suspected or proven *E. faecium* blood culture. Still, fourteen patients (42.4%) had no adequate treatment for their infection after 24 hours.

**Table 4:** **Antibiotic treatment with vancomycin or teicoplanin in patients with *E. faecium *BSI, including mortality rates (*n*** **= 33)**

	**Vancomycine/teicoplanin treatment cases (*n*** **= 33)**					
	**Yes**				**No**	
	**Empirical**	**Mortality**	**Therapeutic**	**Mortality**		**Mortality**
**At time of blood culture withdrawal**	3	2/3	1^*^	0	29	11/29
	(9.1%)	(66.7%)	(3%)	(0%)	(87.9%)	(37.9%)
**After 24 hrs**	4	3/4	13 + 2^*^	5/15	14	5/14
	(12.1%)	(75%)	(45.5%)	(33.3%)	(42.4%)	(35.7%)

*Because of coagulase negative staphylococci.

Additional statistical analyses were performed on patients with an *E. faecium* BSI (cases) to determine additional risk factors for mortality. Only the numbers of blood cultures were found to be statistically significant for mortality at 7 days, with significant trend effect in case of more positive blood cultures. (Additional file 1) None of the other demographic, clinical or microbiologic factors listed in Tables 1 and 2 (e.g. neutropenia, mucositis, glycopeptide treatment) were found to be additional risk factors.

### Multivariable regression analysis and prediction modeling

Variables included in the multivariate regression analyses are shown in Table 5. Independent risk factors for an *E. faecium* BSI are colonization with *E. faecium* 30 days prior to blood culture (OR 5.71; CI 1.7-18.7), combination of neutropenia and abdominal focus (4.37; 1.4-13.4), age > 58 years (4.01; 1.3-12.5), hospital stay prior to blood culture > 14 days (3.55; 0.98-12.9) and CRP (C-reactive protein) level >125 mg/L (4.37; 1.1-10.2).

**Table 5:** **Multivariate logistic regression analyses: risk factors associated with an *E. faecium *BSI (*n*** **= 33)**

**Variables tested**	**B**	***p***	**OR**	**[95% CI]**
A. Colonization with *E. faecium* 30 days prior to blood culture	1.742	0.004	5.71	[1.7–18.7]
B. Neutropenia and abdominal focus^*^	1.474	0.010	4.37	[1.4–13.4]
C. Age > 58 years	1.390	0.017	4.01	[1.3–12.5]
D. Days of admission prior to blood culture > 14 days	1.267	0.054	3.56	[0.98–12.9]
E. CRP >125 mg/L	1.216	0.032	4.37	[1.1–10.2]

^*^Abdominal pain and/or diarrhea. B = regression coefficient. *Pp*-value. *OR* Odds ratio. *95% CI* 95% confidence interval.

Subsequently these independent risk factors were used in order to develop the prediction model. A subset of this prediction model is shown in Table 6. Hereby the formula e^β0+c+ β1X1+…βkXk^ / 1 + e ^β0+c+ β1X1+…βkXk^ was used, whereas β was deduced from the multivariate regression analysis as shown in Table 5. Since five variables were tested and used in this model, a total of 32 outcomes are possible. If a patient has all the five variables at the moment of blood culture withdrawal, the risk of an *E. faecium* BSI is 47.5%. If a patient has none of the variables the risk is close to zero. In clinical decision making the clinician can fill in the variables; 0 for a negative and 1 for a positive score and thereby deduce the risk of *E. faecium* BSI. (All 32 variables and probabilities are available in an Additional file 1).

**Table 6:** **Prediction model to determine the risk of *E. faecium *BSI (subset)**

**A**	**B**	**C**	**D**	**E**	**Probability**
1	1	1	1	1	47.5
1	1	1	1	0	21.2
1	1	1	0	1	20.3
1	1	0	1	1	18.4
1	0	1	1	1	17.2
0	1	1	1	1	13.7
0	0	0	0	0	0.08

For this prediction model the formula e^β0+c+ β1X1+…βkXk^ / 1 + e ^β0+c+ β1X1+…βkXk^ was used, whereas β was deduced from the multivariate regression analysis as shown in Table 5. 0  variable absent, *1*  variable present *A*  Colonization with *E. faecium* 30 days prior to blood culture *B*  Neutropenia and abdominal focus (diarrhea or abdominal pain) *C*  Age over 58 years *D*  Days of admission prior to blood culture more than 14 days *E*  CRP >125 mg/L.

## Discussion

Nowadays *E. faecium* has become an emerging and challenging pathogen in hospitals and even more has replaced *E. faecalis* as the predominant enterococcus species [8]. The increase of *E. faecium* BSIs in our study are in line with the numbers of a recent EARSS (European Antimicrobial Resistance Surveillance System) study, in which *E. faecium* increased most significant in BSIs compared to other major pathogens [15].

All *E. faecium* strains from the blood cultures in our study belonged to the HA/CC-17 strains. They were all amoxicillin (ampicillin) resistant and insertion sequence 16 (*IS*16) positive, which is a marker for these strains [16]. HA/CC-17 strains seem to be successful in acquiring accessory virulence and antibiotic genes and therefore might set the stage for VRE [17]. In vancomycin resistant *E. faecium* infections, adequate treatment of serious infections becomes limited. Although some novel antimicrobials such as linezolid and daptomycin have been developed, these also have their limitations; moreover resistance to these antimicrobials has already been described [18].

In line with previous studies prior colonization with HA *E. faecium* showed to be an independent risk factor for *E. faecium* BSI [19,20]. This study showed that the majority of patients (63.6%) were first colonized prior to the development of *E. faecium* BSI; moreover it seemed to be the most important/significant independent risk factor for *E. faecium* BSI in our study. It is important to keep in mind that multiple swabs might be needed to detect the majority of carriers [21] and *E. faecium* can persist for a long period [22] which is also seen in our study. Environmental contamination and person-to-person spread are factors contributing to the acquisition of *E. faecium*[23,24]. *Enterococcus* spp. are quite tenacious and may survive for more than 4 months under dry conditions [25]. Therefore standard hygiene (e.g. hand hygiene) and appropriate infection-control measures (e.g. risk surface disinfection) are essential.

Neutropenia and abdominal focus (diarrhea and/or abdominal pain) were also associated with *E. faecium* BSI. Because these variables seem to be related to each other, as they individually excluded each other in regression analysis, the two variables were combined. The extensive chemo- and transplantation therapy the patients receive is often associated with neutropenia and diarrhea [26]. In case of severe neutropenia or chemotherapy induced diarrhea which can be seen as injury of the mucosal barrier, *E. faecium* has the opportunity to enter the bloodstream.

Subsequently we expected mucositis, which relates more to the oral toxicity of chemotherapy, to be an associated variable. Kuehnert et al. showed that the risk of VRE BSI increased with increasingly severe mucositis [27]. In contrast, Worth et al. didn’t find mucositis to be associated with *E. faecium* infection; however it hadn’t a well-validated mucositis severity index [28]. Perhaps a more validated mucositis stratification would have shown other results in our study.

CRP level and fever as infection parameters were both found to be significant. However, they individually excluded each other in the regression analysis. Therefore we chose to include CRP level in our model as it is a more objective parameter. Especially in these haematology patients, fever can be aspecifically related to for example drug fever or inflammation like mucositis.

Not many studies have identified age to be an independent risk factor. However the majority of the patients with *E. faecium* infections in the studies are at higher age (50–70 years) and these studies included a more specific control group [11,29,30] whereas we choose a random selection representative for the total population of the haematology ward during the study period.

Since *E. faecium* is considered to be a nosocomial pathogen, a prolonged hospital length of stay as a predictor in *E. faecium* bacteremia is as we expected. For VRE as a multi-resistant pathogen it is clear it is associated with a longer hospital length of stay. Though also for vancomycin-susceptible (VSE), but ampicillin resistant *E. faecium* (ARE) as in our study, this association had been shown [31,32].

Another risk factor often associated with *E. faecium* infection is previous antibiotic use [30]. Moreover, numbers of enterococci in SDD increases since they are not covered [33]. We haven’t found a strong association between antibiotic use and an *E. faecium* BSI, since the majority of both patient groups received SDD.

Additional analysis between patients with and without an *E. faecium* BSI did not result in additional risk factors for mortality besides the total number of positive *E. faecium* blood cultures. However numbers were often too small to perform adequate statistical analyses between the two groups.

This study has some limitations. Firstly, the data was retrospectively gathered. Although both stored-hard copy and electronic records were reviewed, for certain clinical parameters precise monitoring was difficult. Secondly, this is a single-centre study whereas local epidemiological variables and infection prevention measures must be considered. Thirtly, from January 2007 surveillance cultures were screened for meropenem and high level gentamicin resistant *E. faecium*. The reason for this was an increase in *E. faecium* of which the major part was high level gentamicin resistant in our hospital from that time period. An unknown number of *E. faecium* of gentamicin susceptible surveillance cultures have been missed during this period. However, we still detected some gentamicin low level resistant *E. faecium* (5/200 patients ~5%) from that time period. From February 2011 we use 2 mg/L gentamicin in our BMEG screening agars instead of 128 mg/L. Hereby we see an increase of ~30% due to low level gentamicin *E. faecium* in the haematology ward for the period February 2011 – July 2013. However, there seems to be a shift again from 2012, whereas gentamicin high level *E. faecium,* accounts for up to 80% of the HA *E. faecium* both in screening cultures as well as in blood cultures for the period February 2012 – July 2013. This should be taken into account considering results of *E. faecium* colonization in our study. It is difficult to assess the implication of this limitation on the prediction model with respect to the odds ratio. Moreover, patients can have several *E. faecium* strains in their surveillance cultures as well as in blood cultures. Finally the majority of our control group had blood cultures with ‘no growths’. This might have several reasons, for example patients could have had fever due to the malignancy or drug fever or inflammation because of mucositis. It could also partially be explained by the fact patients received SDD. One can state that these patients had a lower degree of illness, compared to patients with an *E. faecium* blood culture. However, retrospective circumstances for both groups were equal. Both groups had the same grounds to obtain a blood culture; neutropenic fever or other clinical signs of infection. Also for the purpose of the study, a prediction model in order to decide whether or not add glycopeptide to the empirical antibiotic treatment at the moment a blood culture is drawn, we choose to select this group of patients as controls.

In conclusion this study demonstrated that colonization with HA *E. faecium* 30 days prior to blood culture, combination of neutropenia and abdominal focus, age > 58 years, hospital stay prior to blood culture > 14 days and CRP level >125 mg/L are independent risk factors for *E. faecium* BSI. In agreement with previous studies, this study showed that *E. faecium* infections can cause severe infections and are associated with high mortality rates in patients with haematologic malignancies [10,34]. Thereby risk stratification becomes necessary in those haematology patients at high risk. Using a prediction model for risk stratification as designed in this study, antibiotic stewardship in terms of prudent use of glycopeptides becomes possible. Together with infection control measures this might be helpful controlling further increase of VRE. The prediction model in this study is based on one specific haematology ward, though it would be worthwhile to verify this prediction model in a prospective multicenter study.

## Competing interest

The authors declare that they have no competing interests.

## Authors’ contributions

XZ has contributed to the conception and design of the study, gathered laboratory and clinical data, analyzed the data and drafted the original article. JA contributed to the conception and design of the study, gathered laboratory data and revised the article. LS contributed to the conception and design of the study and critically revised the article. AF critically revised the article. All authors read and approved the final manuscript.

## Supplementary Material

Additional file 1: Table S1 Association between numbers of *E. faecium* blood cultures and mortality in patients with an *E. faecium* BSI/cases (*n*=33). **Table S2.** Complete prediction model to determine the risk of *E. faecium* BSI. Click here for file
