# Supplementary material for: Algorithm for pre-emptive glycopeptide treatment in patients with haematologic malignancies and an Enterococcus faecium bloodstream infection
Source: Antimicrob Resist Infect Control. 2013 Sep 11;2:24. doi: 10.1186/2047-2994-2-24 (PMC3856451; doi:10.1186/2047-2994-2-24)
Supplement: Additional file 1: Table S1 — Association between numbers of E. faecium blood cultures and mortality in patients with an E. faecium BSI/cases (n=33). Table S2. Complete prediction model to determine the risk of E. faecium BSI. [file 2047-2994-2-24-S1.doc]

Additional supplements

**Table S1**

Association between numbers of *E. faecium* blood cultures and mortality in patients with an *E. faecium* BSI/cases (*n*=33)

| **Mortality at 7 days** | **Yes (*n*=10)** | **No (*n*=23)** | ***p*-value** |
| --- | --- | --- | --- |
| Numbers of *E. faecium* blood cultures, median (range) | 3.5 (1-10) | 1 (1-12) | 0.05 |
| More than one *E. faecium* blood culture | 9 (90%) | 10 (43.5%) | 0.02 |
| More than two *E. faecium* blood cultures | 7 (70%) | 4 (17.4%) | 0.006 |
|  |  |  |  |
| **Mortality at 30 days** | **Yes (*n*=13)** | **No (*n*=20)** | ***p*-value** |
| Numbers of *E. faecium* blood cultures, median (range) | 3.0 (1-10) | 1.5 (1-12) | 0.127 |
| More than two *E. faecium* blood cultures | 7 (53.8%) | 4 (20%) | 0.065 |

**Table S2**

Complete prediction model to determine the risk of *E. faecium* BSI

| A | B | C | D | E | Probability |
| --- | --- | --- | --- | --- | --- |
| 1 | 1 | 1 | 1 | 1 | 47.5 |
| 1 | 1 | 0 | 1 | 1 | 18.4 |
| 1 | 0 | 1 | 1 | 1 | 17.2 |
| 0 | 1 | 1 | 1 | 1 | 13.7 |
| 1 | 1 | 1 | 0 | 1 | 20.3 |
| 1 | 1 | 1 | 1 | 0 | 21.2 |
| 1 | 0 | 0 | 1 | 1 | 4.9 |
| 0 | 1 | 0 | 1 | 1 | 3.8 |
| 1 | 1 | 0 | 0 | 1 | 6.0 |
| 0 | 0 | 1 | 1 | 1 | 3.5 |
| 1 | 1 | 0 | 1 | 0 | 6.3 |
| 1 | 0 | 1 | 0 | 1 | 5.5 |
| 0 | 1 | 1 | 0 | 1 | 4.3 |
| 1 | 0 | 1 | 1 | 0 | 5.8 |
| 0 | 1 | 1 | 1 | 0 | 4.5 |
| 1 | 1 | 1 | 0 | 0 | 7.0 |
| 0 | 0 | 0 | 1 | 1 | 0.9 |
| 1 | 0 | 0 | 0 | 1 | 1.4 |
| 0 | 1 | 0 | 0 | 1 | 1.1 |
| 1 | 0 | 0 | 1 | 0 | 1.5 |
| 0 | 1 | 0 | 1 | 0 | 1.2 |
| 0 | 0 | 1 | 0 | 1 | 1.0 |
| 1 | 1 | 0 | 0 | 0 | 1.8 |
| 0 | 0 | 1 | 1 | 0 | 1.1 |
| 1 | 0 | 1 | 0 | 0 | 1.7 |
| 0 | 1 | 1 | 0 | 0 | 1.3 |
| 0 | 0 | 0 | 0 | 1 | 0.25 |
| 0 | 0 | 0 | 1 | 0 | 0.27 |
| 0 | 1 | 0 | 0 | 0 | 0.33 |
| 1 | 0 | 0 | 0 | 0 | 0.43 |
| 0 | 0 | 1 | 0 | 0 | 0.30 |
| 0 | 0 | 0 | 0 | 0 | 0.08 |

For this prediction model the formula e^β0+c+ β1X1+…βkXk^ / 1+e ^β0+c+ β1X1+…βkXk^  was used, whereas β was deduced from the multivariate regression analysis as shown in table 5. 0 = variable absent, 1 = variable present

A= Colonization with *E. faecium* 30 days prior to blood culture B= Neutropenia and abdominal focus (diarrhea or abdominal pain) C= Age over 58 years D= Days of admission prior to blood culture more than 14 days E= CRP >125mg/L
